# Study on the Trend of Cervical Cancer Inpatient Costs and Its Influencing Factors in Economically Underdeveloped Areas of China, 2019–2023: An Analysis in Gansu Province

**DOI:** 10.3390/healthcare13212663

**Published:** 2025-10-22

**Authors:** Xi Chen, Yinan Yang, Yan Li, Jiaxian Zhou, Dan Wang, Yanxia Zhang, Jie Lu, Xiaobin Hu

**Affiliations:** 1 Institute of Epidemiology and Health Statistics, School of Public Health, Lanzhou University, Lanzhou 730000, China; 2 Department of Pediatrics, The Fifth Affiliated Hospital, Sun Yat-sen University, Zhuhai 519000, China; 3 Health Statistics Information Center of Gansu Province, Lanzhou 730000, China

**Keywords:** cervical cancer, inpatient costs, economic burden, influencing factors, random forest

## Abstract

**Background**: Comprehensive data on the economic burden of cervical cancer treatment remain scarce in China’s less developed regions, necessitating this study on hospitalization costs and expenditure trends in these areas. **Methods**: Employing a multi-stage stratified cluster sampling approach, this study enrolled 10,070 cervical cancer inpatients from 72 healthcare facilities in Gansu Province. Clinical and expenditure data were extracted from hospital information systems. Rank sum tests and Spearman correlation analyses were performed for univariate assessment, while quantile regression and random forest models were applied to identify determinant factors. **Results**: From 2019 to 2023, the average hospitalization duration for cervical cancer patients in Gansu Province was 16.12 days, with an average hospitalization cost of USD 3862.08 (2023 constant prices, converted from CNY at 1:7.0467). During these five years, the average inpatient costs per hospitalization increased from USD 3473.45 to USD 4202.57, and the average daily hospitalization cost rose from USD 230.53 to USD 241.77. The average drug cost decreased from USD 769.06 to USD 640.16. The main factors influencing hospitalization costs included the length of hospital stay, whether cervical cancer surgery was performed, hospital type, hospital level, and the proportion of medications. **Conclusions**: Our findings indicate that cervical cancer is a considerable economic burden on both families and society. This highlights the need to control the length of hospital stay and optimize the allocation of medical resources, in addition to strengthening cervical cancer screening and HPV vaccination in underdeveloped areas, in order to enhance the efficiency of prevention and treatment and ensure medical equity.

## 1. Introduction

Cervical cancer ranks as the fourth most common cancer and the fourth leading cause of cancer-related deaths among women worldwide. In 2022, there were 661,000 new cervical cancer cases and 348,000 deaths globally [1]. China accounted for 22% of the global burden, with 150,700 new cases and 55,700 deaths reported in the same year [2]. Cervical cancer has been labeled as a “disease of the poor,” as its incidence strongly correlates with the national Human Development Index (HDI) [3]. Countries with high HDI (≥0.8) reported an annual age-standardized incidence rate of 9.6 cases per 100,000 women, compared to 26.7 cases per 100,000 women in low-HDI countries [4]. Moreover, the mean age at cervical cancer diagnosis in China has decreased by 5–10 years compared with data prior to 2000, reflecting a trend toward younger patients [5]. These findings underscore the rising global burden of cervical cancer, especially in underdeveloped regions, where it poses a serious threat to women’s health and quality of life.

For patients with early-stage cervical cancer, with the advances in radical hysterectomy combined with lymph node dissection, the 5-year recurrence-free survival rate has exceeded 90%, and the use of combination targeted therapy and immune checkpoint inhibitors has further prolonged the survival of patients with advanced cervical cancer [6,7]. However, advances in diagnostic and therapeutic tools, including the introduction of high-acquisition-cost therapies, have significantly increased overall treatment expenditures compared with traditional regimens, even when survival benefits are observed [8]. A national retrospective cross-sectional analysis in the United States showed that cervical cancer not only leads to a substantial increase in patients’ personal healthcare expenditures, but also may indirectly affect the family’s finances through missed work and long-term health problems [9]. In addition, according to a multinational multicenter study, the global macroeconomic costs of cervical cancer are predicted to be as high as 682 billion international dollars (Purchasing Power Parity, PPP, approximately USD 716 billion) between 2020 and 2050, with the burden being particularly significant in low-and middle-income countries [10]. In China, a nationwide multicenter cross-sectional survey reported that patients with stage IB and above faced catastrophic health expenditures regardless of insurance status, with out-of-pocket costs reaching 1.78–3.59 times the annual disposable income per capita among rural uninsured residents, compared with 0.50–0.83 times for urban insured populations [11]. Similarly, a retrospective case series study in Henan Province revealed that the average annual cost per cervical cancer patient was 1.7 times the provincial Gross Domestic Product (GDP) per capita and 4.0 times the per capita household disposable income [12]. These findings underscore the substantial financial burden imposed by cervical cancer and highlight the importance of region-specific cost analyses.

“Economically underdeveloped regions” are defined as areas with low economic development, a monotonous industrial structure, low per capita disposable income, stagnated development, and limited openness to the outside world [13]. Previous nationwide studies in China suggest that regional economic conditions significantly shape hospitalization costs [11]. However, underdeveloped regions in northwest China remain underrepresented in the literature, despite their markedly lower Healthcare Resource Allocation. From a health economics perspective, research in this area is particularly important, as underdeveloped regions are more vulnerable to catastrophic health expenditures and inequities in healthcare financing. A systematic review and meta-analysis indicated that the CHE rate among cancer patients was 67.9% in low-HDI regions, compared with only 23.4% in very high-HDI regions [14].

Given the aforementioned information, it is crucial to investigate the economic burden of cervical cancer in less developed regions of China. To fill this research gap, we conducted a study in Gansu Province in northwestern China. The region has a resident population of 24.58 million and a GDP of CNY 1300.29 billion, which ranks fifth from the bottom in China. Additionally, in 2023, Gansu’s per capita total health expenditure was only CNY 5185.32, markedly lower than the national average of CNY 6425.32, and the province had just 8.97 health technicians per 1000 population, far fewer than Beijing’s 14.31. Moreover, by the end of 2024, China’s urbanization rate reached 67%, while Gansu’s was only 56.83%, further limiting equitable healthcare access [15]. Cervical cancer prevention and control in Gansu faces additional challenges, including low screening coverage, limited HPV vaccination uptake, and pronounced urban–rural disparities [16]. The objective of this study was to describe the composition and trends of cervical cancer hospitalization costs in Gansu Province between 2019 and 2023. In addition, we explored the factors affecting the average inpatient costs per hospitalization and the daily hospitalization cost of cervical cancer patients and the importance of these factors. The refined analysis of cervical cancer hospitalization costs may provide essential cost data for future studies to assess the cost-effectiveness of cervical cancer prevention and control strategies tailored to underdeveloped areas. Our findings could also help optimize the allocation of health resources and inform policymaking.

## 2. Materials and Methods

### 2.1. Data Source

In this study, a multistage stratified cluster random sampling method was adopted. In the first stage, 1/2 of the entire number of local institutions from general hospitals and Chinese medicine hospitals were selected as samples through simple random sampling, and for specialty hospitals, 1 institution was selected from each category according to specialty categories. In the second stage, Pingliang, Tianshui, Wuwei, Zhangye and Dingxi were selected as sample cities based on the regional economic level and geographic distribution characteristics of 14 cities and states in Gansu Province. The sampling principles of municipal healthcare institutions were consistent with those of provincial institutions. In the third stage, one district and two counties, totaling 15 districts (counties), were selected according to the regional characteristics and urban–rural differences of the sample cities, and their healthcare institutions were selected in the same way as the provincial institutions. In the end, a total of 72 healthcare organizations were included. A detailed flowchart of the selection process is shown in Supplementary Figure S1.

Data for our study were collected from the hospital information system (HIS) of each sample institution from 2019 to 2023 and included general demographic information (age, gender, insurance status, disease diagnosis, time of admission and discharge, length of hospital stay, comorbidities) and inpatient hospitalization costs (including different cost components). Comorbidities were identified using the ICD-10 coding algorithm translated by Elixhauser [17]. The number of comorbid conditions was counted and categorized (0, 1, 2, ≥3), This count variable was then included in the regression models as an indicator of overall comorbidity burden. The comorbidity categories identified included cardiovascular, neurological, endocrine, pulmonary, psychiatric diseases, and neoplasms, with corresponding ICD-10 codes provided in Supplementary Table S1. The present study screened hospitalizations for cervical cancer at various institutions based on the International Classification of Diseases, 10th edition (ICD-10) code C53.

Each observation in this study represents a single hospitalization, and repeated admissions for the same patient could not be reliably tracked due to the limitations of the HIS data. Therefore, the unit of analysis in this study is each hospitalization, and all analyses of inpatient costs are based on individual hospital stays.

### 2.2. Inpatient Cost Components

Hospitalization costs in China are predefined by the hospital information system (HIS). In the HIS, inpatient costs are routinely categorized into seven components: treatment costs, medication costs, general medical service costs, examination costs, surgery costs, nursing costs, and other costs. Medication costs include Western medicine costs and Chinese medicine costs. General medical service fees include bed fees and consultation fees. Examination fees include laboratory test fees and medical imaging fees. Other expenses refer to miscellaneous expenses not categorized under the above six items. treatment cost includes therapeutic procedures excluding surgery, nursing, or general medical services. Following China’s national rules for inpatient charge items and the National Health Commission’s health information data element standards [18]. The proportion of medications denotes the percentage of medication costs in relation to the total inpatient costs.

### 2.3. Data Pre-Processing

Patient information and medical records were anonymized and de-identified prior to analysis. The completeness and authenticity of the data were verified to ensure the accuracy of the study data. After logical correction, non-compliant hospitalized patients were excluded, including those with (1) a primary diagnosis of non-cervical cancer (ICD-10 code not C53), (2) hospitalization length < 1 day or incomplete information on related costs, and (3) extreme values of hospitalization costs. A total of 10,070 medical records of patients hospitalized for cervical cancer were ultimately collected for this study. In order to eliminate the interference of price factors on the hospitalization costs of cervical cancer patients in different years, the consumer price index (CPI) of Gansu Province in 2023 was used as the base year to discount the costs on a comparable basis, and all the costs in this study were uniformly expressed in Chinese Yuan (CNY) in 2023, and have been adjusted accordingly when necessary. Economic data such as Consumer Price Index (CPI), disposable income per capita, and the annual average exchange rate of CNY to USD in 2023 (1:7.0467) are derived from the National Bureau of Statistics of China.

### 2.4. Statistical Analysis

Database construction, cleaning, and statistical analysis were performed using Stata 17.0 and R 4.2.1 software. Categorical variables were expressed as frequency (percentage) {n(q%)} and continuous variables as mean ± standard deviation (SD). To enhance transparency, we also reported medians with interquartile ranges (IQR, P25–P75) for hospitalization costs and lengths of stay. To assess distributional assumptions, we examined histograms and Q–Q plots of hospitalization costs and length of stay. Both variables showed skewed distributions in Supplementary Figure S2, supporting the use of non-parametric tests in univariable analyses. Given the skewed distribution of hospitalization costs, non-parametric tests were applied: the Mann–Whitney U test for two-group comparisons, the Kruskal–Wallis H test for multiple groups, and Spearman rank correlation for associations with age, length of stay, and medication proportion. A two-sided *p* < 0.05 was considered statistically significant.

To further investigate determinants of hospitalization costs, we included covariates selected based on clinical relevance and previous literature into multivariable models, regardless of their univariable significance. Hospitalization costs were log-transformed and modeled using both quantile regression and random forest (RF). Quantile regression was conducted at the 10th, 50th, and 90th percentiles, consistent with prior studies [19], to capture effects of covariates across low, median, and high cost levels. RF, an ensemble machine learning method [20], was applied to identify and rank the importance of predictors [21].

In this study, the dataset from 2019 to 2023 is randomly divided into training and test sets in the ratio of 7:3. By default, the number of generated trees (ntree) was kept at 500, and the best mtry value was determined by automatic hyperparameter optimization in R. The number of variables used in each split (mtry) was set to 4. The variable assignments are shown in Supplementary Table S2, and all statistical analyses with *p* < 0.05 were considered statistically significant.

## 3. Results

### 3.1. Demographic Characteristics and Univariate Analysis

This study included 10,070 cervical cancer inpatients with a mean age of 53.3 ± 10.8 years and a mean hospital stay of 16.12 ± 14.49 days, and the median (IQR) was 12 (7–19) days. Supplementary Table S3 presents the basic characteristics of patients in different age groups. The payment methods were categorized as Medical Insurance Reimbursement or Out of pocket. Medical Insurance Reimbursement accounted for 89.87% (9050 cases). Out-of-pocket payments represented 10.13% (1020 cases). Nearly half (48.41%) of patients underwent surgical treatment, with hospitalization costs significantly higher than those of non-surgical patients. Basic characteristics of cervical cancer surgery patients and non-surgery procedure patients are presented in Supplementary Table S4. By administrative classification of healthcare institutions, over 95% of cases were treated at provincial or municipal-level facilities. Among the included cases, 59.12% received care in general hospitals and maternal/child health hospitals, with 37.65% treated in specialized hospitals (including cancer-specialized hospitals), while fewer than 4% sought treatment at Traditional Chinese Medicine hospitals.

Univariate analysis showed that the differences in the cost per hospitalization for cervical cancer patients were statistically significant (*p* < 0.01) across age, number of days in the hospital, whether or not they were operated on, number of comorbidities, method of payment, type of institution, and level of institution. The results of the analysis are presented in Table 1.

### 3.2. Component of Inpatient Costs for CERVICAL Cancer

**Figure 1:**
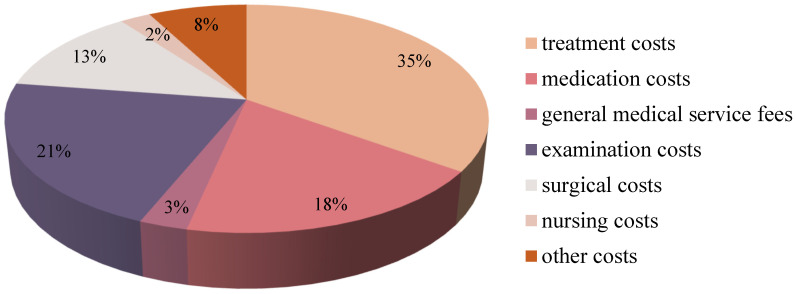
Components of inpatient costs of cervical cancer patients in Gansu Province, China, from 2019 to 2023.

**Figure 2:**
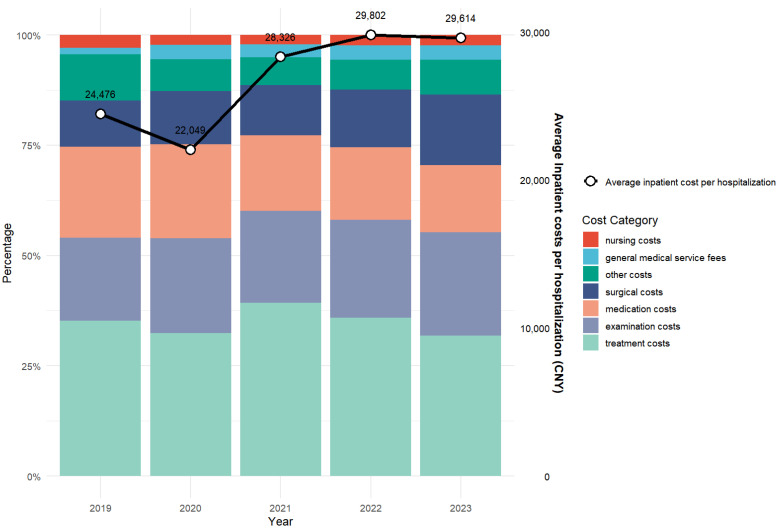
Inpatient Cost Components for Cervical Cancer in Gansu Province, China, from 2019 to 2023.

From 2019 to 2023, the average hospitalization cost for cervical cancer patients in Gansu Province was CNY 27,214.71 (USD 3862.08). Analyzing the expenditure in detail, the treatment cost was CNY 9518.25 (USD 1350.70, 35.00%) and the examination cost was CNY 5775.41 (USD 819.62, 21.22%), which is a major component of the hospitalization cost. This was followed by CNY 5005.93 (USD 710.48, 18.39%) for drugs, CNY 3390.11 (USD 481.09, 12.46%) for surgery, and CNY 754.87 (USD 107.12, 2.77%) for general medical services. The lowest percentage of nursing costs was CNY 660.06 (USD 93.67, 2.43%), with other costs reaching CNY 2112.82 (USD 299.83, 7.76%), as shown in Figure 1 and Figure 2.

### 3.3. The Trend of Inpatient Costs for Cervical Cancer

As shown in Table 2, between 2019 and 2023, the inpatient costs per hospitalization for cervical cancer in Gansu Province increased significantly from CNY 24,476.40 ± 24,365.62 (median [IQR]: 13,631.68 [7619.34–32,908.67]) to CNY 29,614.44 ± 28,289.92 (median [IQR]: 23,626.68 [7660.61–37,555.98]) (*p* < 0.05, Kruskal–Wallis test), with an average five-year growth rate of 11.19%. Similarly, average out-of-pocket costs rose from CNY 3960.03 ± 6258.97 (median [IQR]: 1419.35 [325.93–4761.36]) in 2019 to CNY 8671.20 ± 8316.12 (median [IQR]: 6304.84 [2341.85–12,588.07]) in 2023 (*p* < 0.05), despite a slight decrease between 2022 (CNY 10,642.45) and 2023, resulting in a cumulative increase of 44.25%. In contrast, the average daily hospitalization cost showed a modest, non-significant increase from CNY 1624.46 ± 944.95 (median [IQR]: 1478.27 [1054.42–1933.07]) to CNY 1703.68 ± 926.18 (median [IQR]: 1653.03 [1145.59–2087.17]) (*p* < 0.05), with an average growth rate of 0.98%.

### 3.4. Quantile Regression Analysis of Inpatient Costs

The quantile regression results indicated that length of hospital stay, hospital level, hospital type, cervical cancer surgery, and payment method were significantly associated with the 10th, 50th, and 90th percentiles of inpatient costs per hospitalization (*p* < 0.05). Furthermore, length of hospital stay, hospital level, hospital type, number of comorbidities, and cervical cancer surgery emerged as significant determinants across all three percentiles of average daily hospitalization costs (*p* < 0.05). The quantile regression pseudo R2 for the 10th, 50th and 90th percentiles of the cost per hospitalization were 0.501, 0.585 and 0.536, respectively, while the average daily hospitalization cost was 0.297, 0.208 and 0.122.

Length of hospitalization, hospital type, hospital level, proportion of medications, cervical cancer surgery, comorbidities, payment method, and patient age were significantly associated with inpatient costs per hospitalization and average daily hospitalization costs across multiple percentiles (*p* < 0.05 or *p* < 0.001). With respect to age, patients aged 40 years or above showed higher inpatient costs per hospitalization at the 10th percentile compared to those under 40 (*p* < 0.05). Out-of-pocket payments were negatively correlated with both cost measures at the 10th and 50th percentiles, though a slight positive association was observed at the 90th percentile for per-hospitalization costs (*p* < 0.05). Compared to public hospitals, private hospitals exhibited lower costs at the 10th percentile but higher costs at the 90th percentile for inpatient expenses per hospitalization (*p* < 0.001). A clear gradient was observed by hospital level: compared with provincial hospitals, inpatient costs per hospitalization and average daily hospitalization costs were significantly lower at all percentiles in municipal-, district-, and county-level hospitals (*p* < 0.001). In terms of hospital type, Traditional Chinese Medicine hospitals consistently had lower costs than general hospitals across percentiles, while Maternal and Child Healthcare hospitals showed significantly reduced costs at the 50th percentile (*p* < 0.05). Specialized hospitals showed lower inpatient costs per hospitalization at the 10th and 50th percentiles compared with general hospitals, but significantly higher average daily hospitalization costs at the 90th percentile (*p* < 0.001). The number of comorbidities was positively linked to hospitalization costs, with effect sizes increasing alongside comorbidity count throughout all percentiles for both cost types (*p* < 0.05). Longer hospital stays were consistently associated with elevated inpatient costs per hospitalization across all percentiles (*p* < 0.001). A higher proportion of medication expenses correlated with increased per-hospitalization costs at lower percentiles, but decreased average daily costs at higher percentiles (*p* < 0.001). Cervical cancer surgery was consistently and positively associated with both inpatient costs per hospitalization and average daily hospitalization costs across all percentiles (*p* < 0.001). Complete numerical results for all percentiles are provided in Supplementary Tables S5–S10. A summary of key findings is presented in Table 3.

### 3.5. Random Forest Analysis of Inpatient Costs

In the Random Forest (RF) model, the 2019–2023 dataset is randomly divided into a training set and a test set at a ratio of 7:3. The number of trees generated (ntree) was set to 500 by default, and the number of variables used in each split (mtry) was set to 4. For the average hospitalization cost, the root mean square error (RMSE) of the random forest model was 0.362, and the coefficient of determination R2 was 0.885; and for the average daily hospitalization cost, the root mean square error was 0.361, and the coefficient of determination R2 was 0.566.

**Figure 3:**
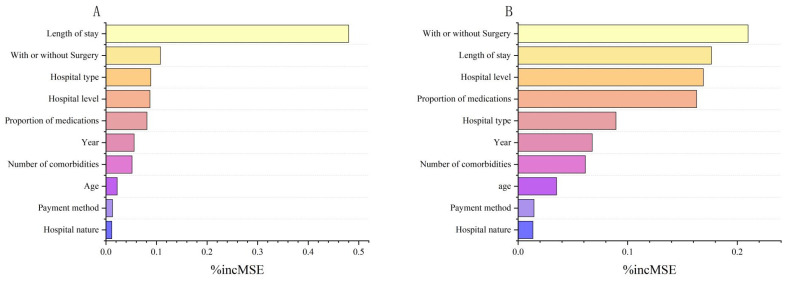
Analysis of the importance of factors influencing inpatient costs for patients with cervical cancer. (**A**) Ranking of the importance of factors influencing inpatient costs per hospitalization for patients with cervical cancer. (**B**) Ranking of the importance of factors influencing daily inpatient costs for patients with cervical cancer.

Figure 3 shows the factors influencing inpatient costs in descending order of importance according to the average precision of decline (%IncMSE). The results showed that the five factors that had the greatest impact on the cost per hospitalization were, in order, length of hospital stay, cervical cancer surgery, hospital type, hospital grade, and medication ratio. The five factors that had the greatest impact on the average daily hospitalization cost were, in order, cervical cancer surgery, length of hospital stay, hospital grade, proportion of medications, and hospital type.

## 4. Discussion

This study is the first to systematically examine hospitalization costs and their determinants among cervical cancer patients in Northwest China. Between 2019 and 2023, the average inpatient cost per hospitalization for cervical cancer patients in Gansu Province increased significantly from CNY 24,476.40 (USD 3473.45) (mean ± SD: 24,476.40 ± 24,365.62; median [IQR]: 13,631.68 [7619.34, 32,908.67]) in 2019 to CNY 29,614.44 (USD 4202.57) (mean ± SD: 29,614.44 ± 28,289.92; median [IQR]: 23,626.66 [14,660.61, 37,555.98]) in 2023, with an average annual growth rate of 4.9%. Given the skewed distribution and wide variability in costs (large SDs and IQRs; Supplementary Figure S2), these results should be interpreted with caution. Compared with other regions in China, Gansu Province’s five-year average inpatient costs per hospitalization for cervical cancer were CNY 27,214.71 (USD 3862.08) higher than those reported in northern China CNY 26,642 (USD 3780.75) [22], and lower than the data from the national special survey [23]. Since hospitalization costs and lengths of stay showed a skewed distribution, we reported both mean ± SD and median (IQR) and conducted non-parametric group comparisons (Mann–Whitney U and Kruskal–Wallis tests) to improve transparency and robustness. The cost for patients who did not undergo surgery for cervical cancer in Gansu Province was CNY 21,143.46 (USD 2999.06), which was higher than CNY 15,510.51 (USD 2198.26) in a study from Henan Province [24]. Cervical cancer hospitalization costs in Gansu Province were substantially lower than those reported in high-income countries. In the United States, the median total first-year treatment cost for commercially insured patients reached USD 56,250, with a median hospitalization cost of USD 15,145 [25], while another study estimated the average hospitalization cost at USD 9646 [26]. By contrast, in low-income countries, the burden is often comparable to annual income levels; in Ethiopia, the average hospitalization cost was USD 404.4, equivalent to 86% of per capita annual income [27]. In Gansu Province, however, the per capita disposable income in 2023 was CNY 25,011 (USD 3549.23), while the five-year average inpatient cost per hospitalization for cervical cancer reached CNY 27,214.71 (USD 3861.97). Although hospitalization costs in Gansu Province are relatively low in absolute terms, they still impose a substantial economic burden, accounting for 108.8% of the 2023 per capita disposable income and exceeding the internationally recognized threshold for catastrophic health expenditure [28]. In this study, 70% of patients were covered by the Urban–Rural Resident Basic Medical Insurance, which may not fully cover high-cost treatments such as radiotherapy and targeted drugs, potentially causing financial hardship. These results should be interpreted with caution, given the wide variability in costs (large SDs and IQRs). Given these considerations, it is crucial for Gansu to prioritize including high-value cervical cancer diagnostic and treatment items in medical insurance and to promote free screening, which could help detect cases earlier and reduce overall treatment costs.

The composition of hospitalization costs is important, with treatment, medication, examination and surgery accounting for 79% of the total costs in this study. The relatively high examination costs likely reflect essential diagnostic procedures, such as HPV-DNA testing and pelvic MRI for initial evaluation, as well as advanced imaging like whole-body PET-CT for advanced-stage patients [29]. Gansu Province’s average medication costs per hospitalization decreased from CNY 5418.72 (USD 769.06) to CNY 4511.02 (USD 640.16) between 2019 and 2023. The temporal decrease in drug costs overlapped with the implementation of Volume-based Procurement Policy (VBP), although our study cannot establish causality, the policy significantly reduces drug prices and eases the burden on patients by exchanging volume for price and compressing costs in the distribution chain [30]. Prior studies have indicated that this policy alleviates the financial burden on patients while promoting the utilization of generic drugs and optimizing the structure of healthcare expenditure [31]. Although our study did not directly evaluate VBP, the temporal alignment suggests it may have contributed.

The high-incidence age group of cervical cancer is usually composed of people in perimenopause [32].The median age of cervical cancer patients in this study was 53 years, higher than reported in the United States [33] and South Africa [34]. which may reflect differences in the sophistication of regional cervical cancer screening systems: well-designed screening policies that optimize the age range and frequency can improve early detection rates [35]. In Gansu Province, relatively low screening rates likely contribute to diagnoses at intermediate or advanced stages. Data from 2021 showed that the first-dose HPV vaccination rate for girls aged 9–14 years was less than 1%, and the overall vaccination rate for women aged 9–45 years was only 2.02% [36], which is much lower than that in developed regions of China and international levels [37,38]. Such low coverage is associated with later diagnosis and potentially higher hospitalization costs. A national modeling study provides insights for solving this problem: if the cost-effectiveness threshold of the domestic bivalent vaccine ($26/dose) is implemented, the national immunization planning budget needs to be increased by more than 70%, but in the long term, it can save $179 million per year in the cost of cervical cancer treatment through the reduction of intermediate and late-stage cases [39]. For underdeveloped regions with low vaccination rates, such as Gansu, this provides important insights for vaccine policy design. Our findings highlight the importance of including less developed regions in central government subsidy programs and reducing vaccine unit prices through centralized procurement to lower cervical cancer incidence. Policy support and health education should also promote earlier intervention, increased screening, and wider vaccination coverage, particularly among younger populations, to meet the WHO “90–70–90” targets [40].

Based on the analysis of quantile regression and random forest model, hospitalization cost is mainly associated with four major factors: length of hospital stay, surgical operation, hospital type and grade, and the proportion of medications. Among them, the influence of hospitalization length and hospital grade is consistent with previous studies, while this study further reveals the key role of proportion of medications and hospital type. The average length of hospital stay for cervical cancer patients in Gansu Province was 16.12 days, which was significantly higher than that in other regions [41,42]. The reason for this was related to both the high proportion of surgical patients in the study and the complex treatments such as postoperative adjuvant radiotherapy, in addition to the delay in recovery was due to the insufficient referral capacity of district and county-level medical institutions and the low prevalence of minimally invasive surgery in Gansu Province [43]. These patterns are consistent with the “Inverse Care Law,” which posits that populations with the greatest healthcare needs often have the least access to high-quality services [44].

Drawing on these findings, we propose that two systematic strategies be implemented: First, our quantile regression results showed that inpatient costs in provincial-level hospitals were consistently higher across all percentiles compared with municipal-, district-, and county-level institutions. Accordingly, implementing differentiated reimbursement policies based on hospital type and level could potentially help alleviate the economic burden on patients. Second, both quantile regression and random forest analyses indicated that surgical treatment and the number of comorbidities were strongly associated with inpatient costs, with higher comorbidity counts leading to significantly increased per-case and per-day hospitalization expenses. In this context, accelerating the adoption of Diagnosis-Related Group (DRG) payment reforms and establishing bundled payment standards that incorporate disease type, treatment complexity, and resource utilization may provide stronger incentives for cost control [45]. Third, length of hospital stay was consistently associated with hospitalization costs across all percentiles. Efforts to strengthen the three-tier referral system linking oncology hospitals, general hospitals, and primary care institutions may help optimize resource allocation, enhance primary-level service capacity, and reduce overtreatment, thereby contributing to a more appropriate hospitalization duration. Moreover, our analyses also highlighted the substantial role of the proportion of medications. Reinforcing medication cost control through centralized volume-based procurement and price negotiations, while simultaneously promoting the uptake of generic drugs, could be beneficial for optimizing the healthcare expenditure structure and mitigating the burden of daily medication costs for patients. Finally, our findings also provide evidence on how social determinants—including economic development, healthcare resources, and insurance coverage—affect hospitalization costs in underdeveloped regions.

There are some limitations to this study. A primary limitation is that only the hospitalization costs of patients with a primary diagnosis of cervical cancer were analyzed, and the costs of cervical cancer patients hospitalized for complications or other reasons, in addition to indirect costs, were not covered. Moreover, the study data came from the hospital information system, which included a limited number of variables that did not include other variables that might affect hospitalization costs. Another constraint was that due to data limitations, information about the disease stage was not available in our dataset. Additionally, information on discharge outcomes (e.g., cure, improvement, mortality, or transfer) was not available, preventing analysis of potential relationships between treatment outcomes and hospitalization costs. Finally, our study only reflects the costs of cervical cancer patients in Northwest China. Nonetheless, as the first study to systematically analyze the trend of cervical cancer hospitalization costs and the factors affecting them in Northwest China, our findings may contribute to further understanding of the economic burden posed by cervical cancer and inform the adjustment of health insurance policies.

## 5. Conclusions

This study is the first to systematically report the trends and influencing factors of hospitalization costs for cervical cancer patients in economically underdeveloped areas of Northwest China. Multivariable analyses indicated that the length of hospital stay, surgical procedures performed, hospital type and level, and drug ratio were the main factors affecting hospitalization costs. Our findings highlight that cervical cancer imposes a considerable economic burden on patients’ families and society. To alleviate this burden, it is recommended to reduce unnecessary hospitalization days and to continuously improve the allocation and efficiency of medical resources.

## Figures and Tables

**Table 1:** Basic characteristics of patients and univariate analysis of average hospitalization costs per hospitalization.

Category	n (q%)	Median (Lower Quartile, Upper Quartile) (CNY)	Test Statistic	*p* Value
All cases	10,070	17,276.06 (7459.69, 36,119.08)	-	-
Age (years) [mean (SD)]	53.29 (10.84)	-	10.750	0.013
<40	1125 (11.17)	18,827.59 (8109.91, 34,423.58)	-	-
40–50	2756 (27.23)	19,258.37 (8037.37, 34,868.09)	-	-
51–60	3826 (38.00)	17,607.94 (7322.41, 36,812.16)	-	-
>60	2363 (23.46)	13,777.08 (6780.49, 37,826.75)	-	-
Payment method	-	-	52.377	<0.001
Medical Insurance Reimbursement	9050 (89.87)	18,080.88 (7589.05, 36,958.31)	-	-
Out of pocket	1020 (10.13)	12,227.59 (6291.08, 29,720.19)	-	-
Hospital nature	-	-	20.213	<0.001
Public	9854 (99.14)	17,468.32 (7515.41, 36,255.36)	-	-
Private	216 (0.86)	11,834.28 (4798.46, 29,891.17)	-	-
Hospital level	-	-	1861.241	<0.001
Provincial level	7305 (72.54)	25,431.85 (10,648.20, 40,488.33)	-	-
Municipal level	2172 (21.57)	7318.81 (4024.11, 19,778.09)	-	-
County level	382 (3.79)	5966.87 (3982.08, 10,023.80)	-	-
District level	211 (2.10)	3305.89 (2410.50, 4910.77)	-	-
Hospital type	-	-	647.527	<0.001
General hospital	3225 (32.03)	11,569.07 (5431.14, 34,646.56)	-	-
Traditional Chinese Medicine (TCM) Hospital	326 (3.24)	6637.59 (3340.82, 13,654.98)	-	-
Maternal and Child Healthcare Hospital	2728 (27.09)	27,403.13 (13,165.57, 37,064.01)	-	-
Specialized Hospital	3791 (37.65)	16,100.47 (7976.39, 38,343.65)	-	-
Length of stay (days) [mean (SD)]	16.12 (14.49)	12.00 (7.00, 19.00)	0.8708	<0.001
Proportion of medications (%) [mean (SD)]	22.25 (17.34)	16.95(10.19, 29.79)	−0.1050	<0.001
Number of comorbidities	-	-	1510.945	<0.001
0	2807 (27.87)	8818.37 (4918.76, 18,390.16)	-	-
1	1864 (18.51)	11,733.54 (6664.06, 29,199.61)	-	-
2	1457 (14.47)	20,432.35 (8029.85, 41,793.89)	-	-
≥3	3942 (39.15)	30,237.92 (14,624.45, 44,381.64)	-	-
Cervical cancer surgery	-	-	2150.100	<0.001
No	5194 (51.58)	8496.51 (4967.65, 16,566.18)	-	-
Yes	4876 (48.42)	29,945.38 (18,782.61, 40,169.95)	-	-
Year	-	-	66.905	<0.001
2019	2279 (22.63)	14,641.98 (8184.04, 35,347.66)	-	-
2020	2601 (25.83)	12,940.26 (7065.13, 32,657.75)	-	-
2021	2078 (20.64)	20,566.93 (6924.53, 39,220.86)	-	-
2022	1437 (14.27)	22,293.51 (7552.87, 37,101.65)	-	-
2023	1675 (16.63)	23,626.68 (7660.61, 37,555.98)	-	-

Hospitalization costs per hospitalization is measured in Chinese Yuan (CNY); length of stay is measured in days. Mean ± standard deviation (mean ± SD) and median (lower quartile, upper quartile) are provided for hospitalization costs per hospitalization and proportion of medications.

**Table 2:** Trends of inpatient costs for cervical cancer.

Year	Inpatient Costs per Hospitalization (CNY) ^a^		Annual Change (%) ^b^	Out-of-Pocket Costs (CNY) ^a^		Annual Change (%)	Daily Inpatient Costs (CNY)		Annual Change (%)
	Mean ± SD	Median (P25, P75)		Mean ± SD	Median (P25, P75)		Mean ± SD	Median (P25, P75)	
2019	24,476.40 ± 24,365.62	13,631.68 (7619.34, 32,908.67)	-	3960.03 ± 6258.97	1419.35 (325.93, 4761.36)	-	1624.46 ± 944.95	1478.27 (1054.42, 1933.07)	-
2020	22,048.84 ± 22,569.23	12,319.13 (6726.00, 31,090.18)	−9.92 ^b^	5768.27 ± 9823.62	2878.15 (768.55, 7896.41)	45.66	1608.97 ± 814.20	1556.06 (1028.53, 2015.38)	−0.95
2021	28,325.95 ± 30,853.44	19,764.82 (6654.47, 37,691.25)	28.47	8512.45 ± 17,751.83	4991.06 (1414.67, 10,677.51)	47.57	1652.19 ± 1067.88	1573.53 (1004.25, 2056.84)	2.69
2022	29,802.47 ± 30,684.90	22,182.04 (7515.11, 36,916.14)	5.21	10,642.45 ± 13511.16	7019.16 (2751.68, 13,370.74)	25.02	1633.02 ± 933.83	1570.94 (1040.89, 2082.88)	−1.16
2023	29,614.44 ± 28,289.92	23,626.68 (7660.61, 37,555.98)	−0.63	8671.20 ± 8316.12	6304.84 (2341.85, 12,588.07)	−18.52	1703.68 ± 926.18	1653.03 (1145.59, 2087.17)	4.33
Overall ^c^	27,214.71 ± 28,087.76	16,584.33 (7169.00, 34,944.73)	11.19 ^c^	7103.72 ± 11,900.45	3878.38 (983.75, 9678.33)	44.25	1640.58 ± 936.17	1560.23 (1053.13, 2015.77)	0.98
*p* value	<0.001		-	<0.001		-	<0.001		-

^a^ The mentioned costs are the costs of cervical cancer patients per hospitalization. ^b^ The annual growth rate is the year-over-year growth rate based on the previous year. ^c^ The overall growth rate is the average growth rate from 2019 to 2023.

**Table 3:** Quantile regression analysis of cervical cancer inpatient costs (CNY).

Variables	Inpatient Costs per Hospitalization			Average Daily Hospitalization Costs		
	Q10	Q50	Q90	Q10	Q50	Q90
Age (contrast ≤ 40)						
40–50	0.101 *	0.022	0.022	0.059	0.008	0.008
51–60	0.061 *	0.015	0.007	0.035	0.008	−0.001
>60	0.082 *	0.008	0.026	0.048	0.011	0.025
Payment method (contrast = medical insurance reimbursement)						
Out-of-pocket	−0.162 †	−0.044 *	0.008 *	−0.090 †	−0.053 †	−0.007
Hospital nature (contrast = public)						
Private	−0.262 †	0.034	0.206 †	−0.072	0.049	0.243 †
Hospital level (contrast = provincial level)						
Municipal level	−0.525 †	−0.514 †	−0.299 †	−0.567 †	−0.443 †	−0.328 †
District level	−0.578 †	−0.527 †	−0.694 †	−0.631 †	−0.567 †	−0.714 †
county level	−0.882 †	−1.067 †	−1.185 †	−0.929 †	−0.941 †	−0.856 †
Hospital type (contrast = general hospital)						
Traditional Chinese Medicine (TCM) hospital	−0.479 †	−0.282 †	−0.350 †	−0.227 †	−0.327 †	−0.459 †
Maternal and Child Healthcare Hospital	−0.104 †	−0.159 †	−0.349 †	−0.015	−0.159 †	0.075 *
Specialized Hospital	−0.017 *	−0.066 †	−0.235 †	−0.026	−0.106 †	0.090 †
Number of comorbidities (contrast = 0)						
1	0.065 *	0.096 †	0.147 †	0.031 *	0.067 †	0.115 †
2	0.198 †	0.119 †	0.158 †	0.093 †	0.102 †	0.131 †
≥3	0.304 †	0.165 †	0.177 †	0.129 †	0.106 †	0.169 †
Length of stay	0.046 †	0.052 †	0.053 †	0.004 †	0.001 †	−0.005 †
Proportion of medications	0.758 †	0.356 †	0.051	0.275 †	−0.036	−0.145 †
Cervical cancer surgery (contrast = No)						
Yes	0.729 †	0.698 †	0.521 †	0.395 †	0.292 †	0.092 †
Year (contrast = 2019)						
2020	0.082 *	0.055 †	0.028	0.020	0.075 †	0.039
2021	0.122 †	0.037 *	0.045	0.016	0.037 *	0.042
2022	−0.078 *	−0.035	−0.006	−0.068 *	−0.030	−0.014
2023	0.020 †	0.011	0.009	−0.010	0.019	−0.006
_cons	7.826 †	8.597 †	9.306 †	6.532 †	7.234 †	8.089 †
Pseudo *R*^2^	0.501	0.585	0.536	0.297	0.208	0.122

* *p* < 0.05 and † *p* < 0.001.

## Data Availability

The data presented in this study are available on request from the corresponding author. The data are not publicly available due to privacy and ethical restrictions.

## Supplementary Materials

The following supporting information can be downloaded at https://www.mdpi.com/article/10.3390/healthcare13212663/s1, Table S1: ICD-10 codes for cervical cancer comorbidities; Table S2: Variable assignment of quantile regression and random forest model; Table S3: Basic characteristics of patients in different age groups; Table S4: Basic characteristics of cervical cancer surgery patients and non-surgery procedure patients; Tables S5–Table S10: Quantile Regression Results for Inpatient costs per hospitalization (Q10\Q50\Q90); Quantile Regression Results for Average Daily Inpatient Cost (Q10\Q50\Q90); Figure S1: Institutional selection process flowchart. Figure S2: presents histograms and Q–Q plots for inpatient costs and length of stay in cervical cancer patients.

## References

[1] Bray F., Laversanne M., Sung H., Ferlay J., Siegel R.L., Soerjomataram I., Jemal A. (2024). Global cancer statistics 2022: GLOBOCAN estimates of incidence and mortality worldwide for 36 cancers in 185 countries. CA Cancer J. Clin..

[2] Wang S., Zheng R., Han B., Li L., Chen R., Sun K. (2024). Age distribution of cancer incidence and mortality in China in 2022. China Cancer.

[3] Singh G.K., Azuine R.E., Siahpush M. (2012). Global Inequalities in Cervical Cancer Incidence and Mortality are Linked to Deprivation, Low Socioeconomic Status, and Human Development. Int. J. MCH AIDS.

[4] Arbyn M., Weiderpass E., Bruni L., de Sanjosé S., Saraiya M., Ferlay J., Bray F. (2020). Estimates of incidence and mortality of cervical cancer in 2018: A worldwide analysis. Lancet Glob. Health.

[5] Jinyao W., Nianping Z., Zhiqiang B., Zhenkun W. (2022). Age-period-cohort model analysis of long-term trends in incidence and mortality of cervical cancer in China from 1993 to 2017. Chin. Gen. Pract..

[6] Mayadev J.S., Ke G., Mahantshetty U., Pereira M.D., Tarnawski R., Toita T. (2022). Global challenges of radiotherapy for the treatment of locally advanced cervical cancer. Int. J. Gynecol. Cancer.

[7] Wenzel H.H., Smolders R.G., Beltman J.J., Lambrechts S., Trum H.W., Yigit R., Zusterzeel P.L., Zweemer R.P., Mom C.H., Bekkers R.L. (2020). Survival of patients with early-stage cervical cancer after abdominal or laparoscopic radical hysterectomy: A nationwide cohort study and literature review. Eur. J. Cancer.

[8] Lin Y.T., Wang C., He X.Y., Yao Q.M., Chen J. (2024). Comparative cost-effectiveness of first-line pembrolizumab plus chemotherapy vs. chemotherapy alone in persistent, recurrent, or metastatic cervical cancer. Front. Immunol..

[9] Shah R., Nwankwo C., Kwon Y., Corman S.L. (2020). Economic and humanistic burden of cervical cancer in the United States: Results from a nationally representative survey. J. Women’s Health.

[10] Chen S., Cao Z., Prettner K., Kuhn M., Yang J., Jiao L., Wang Z., Li W., Geldsetzer P., Bärnighausen T. (2023). Estimates and projections of the global economic cost of 29 cancers in 204 countries and territories from 2020 to 2050. JAMA Oncol..

[11] Chen H., Zhao X., Hu S., You T., Xia C., Gao M., Dong M., Qiao Y., Zhao F. (2023). Estimation of economic burden throughout course of cervical squamous intraepithelial lesion and cervical cancer in China: A nationwide multicenter cross-sectional study. Chin. J. Cancer Res..

[12] Gao L., Wu Q., Jia M., Chen H., Zhang S., Liu Y., Prem K., Qian M., Yu H. (2020). The economic burden of cervical cancer from diagnosis to one year after final discharge in Henan Province, China: A retrospective case series study. PLoS ONE.

[13] Yang X., Wang C. (2006). Research on models and types of less-developed region based on natural and human factors. J. Univ. Chin. Acad. Sci..

[14] Doshmangir L., Hasanpoor E., Abou Jaoude G.J., Eshtiagh B., Haghparast-Bidgoli H. (2021). Incidence of Catastrophic Health Expenditure and Its Determinants in Cancer Patients: A Systematic Review and Meta-analysis. Appl. Health Econ. Health Policy.

[15] National Bureau of Statistics of China (2024). China Statistical Yearbook 2024.

[16] Chen Y., Jiang N., Jiao Y., Chen J., Cao A., An J., Dang Y. (2025). Analysis of HPV vaccination and influencing factors among 9-14-year-old girls in underdeveloped areas of northwestern China: A cross-sectional survey report on guardians. Vaccine.

[17] Quan H., Sundararajan V., Halfon P., Fong A., Burnand B., Luthi J.C., Saunders L.D., Beck C.A., Feasby T.E., Ghali W.A. (2005). Coding algorithms for defining comorbidities in ICD-9-CM and ICD-10 administrative data. Med. Care.

[18] Han S., Xiaolin Y., Sheng Y., Wei Z., Shengfa Z., Hong M.F., Lihong L., Yan Z. (2024). Comparative Study of the 2023 and 2011 Versions of Health Information Data Element Standards. J. Med. Inform..

[19] Zhou S., Yang Y., Wang L., Liu H., Wang X., Ouyang C., Pan J., Hu X. (2024). Study on the trend of congenital heart disease inpatient costs and its influencing factors in economically underdeveloped areas of China, 2015–2020: A case study of Gansu Province. Front. Public Health.

[20] Breiman L. (2001). Random forests. Mach. Learn..

[21] Grömping U. (2015). Variable importance in regression models. Wiley Interdiscip. Rev. Comput. Stat..

[22] Wang M., Yin J., Zhu Y., Zuo T., Zhu B., You J., Yang W., Wang H., Qiao Y., Wang Y. (2025). Hospitalization costs for cervical cancer patients in three tertiary hospitals in northern China, 2013–2022: Degree of structural variation and gray relational analysis. Chin. J. Public Health.

[23] Yin X., Xu Y., Man X., Liu L., Jiang Y., Zhao L., Cheng W. (2019). Direct costs of both inpatient and outpatient care for all type cancers: The evidence from Beijing, China. Cancer Med..

[24] Jian W., Yifei F., Yilin H. (2024). Analysis of Hospitalization Expenses and Influencing Factors of Surgical and Non-surgical Cervical Cancer Patients Based on Quantile Regression. Chin. Med. Rec..

[25] Blanco M., Chen L., Melamed A., Tergas A.I., Khoury-Collado F., Hou J.Y., Clair C.M.S., Ananth C.V., Neugut A.I., Hershman D.L. (2021). Cost of care for the initial management of cervical cancer in women with commercial insurance. Am. J. Obstet. Gynecol..

[26] Corman S., Nwankwo C., Kebede N., Shah R. (2019). Inpatient burden of cervical and uterine cancer in the United States. Am. Soc. Clin. Oncol..

[27] Derbie A., Mekonnen D., Nibret E., Misgan E., Maier M., Woldeamanuel Y., Abebe T. (2023). Cervical cancer in Ethiopia: A review of the literature. Cancer Causes Control.

[28] Zhao Y., Atun R., Oldenburg B., McPake B., Tang S., Mercer S.W., Cowling T.E., Sum G., Qin V.M., Lee J.T. (2020). Physical multimorbidity, health service use, and catastrophic health expenditure by socioeconomic groups in China: An analysis of population-based panel data. Lancet Glob. Health.

[29] Cibula D., Raspollini M.R., Planchamp F., Centeno C., Chargari C., Felix A., Fischerová D., Jahnn-Kuch D., Joly F., Kohler C. (2023). ESGO/ESTRO/ESP Guidelines for the management of patients with cervical cancer–Update 2023. Virchows Arch..

[30] Yuan J., Lu Z.K., Xiong X., Jiang B. (2021). Lowering drug prices and enhancing pharmaceutical affordability: An analysis of the national volume-based procurement (NVBP) effect in China. BMJ. Glob. Health.

[31] Wang J., Yang Y., Xu L., Shen Y., Wen X., Mao L., Wang Q., Cui D., Mao Z. (2021). The impact of National Centralized Drug Procurement policy on the use of policy-related original and generic drugs in public medical institutions in China: A difference-in-difference analysis based on national database. medRxiv.

[32] Troìa L., Martone S., Morgante G., Luisi S. (2021). Management of perimenopause disorders: Hormonal treatment. Gynecol. Endocrinol..

[33] Cohen P.A., Jhingran A., Oaknin A., Denny L. (2019). Cervical cancer. Lancet.

[34] Hull R., Mbele M., Makhafola T., Hicks C., Wang S.M., Reis R.M., Mehrotra R., Mkhize-Kwitshana Z., Kibiki G., Bates D.O. (2020). Cervical cancer in low and middle-income countries. Oncol. Lett..

[35] Choi S., Ismail A., Pappas-Gogos G., Boussios S. (2023). HPV and cervical cancer: A review of epidemiology and screening uptake in the UK. Pathogens.

[36] An J., Liu Y., Ma Y., Jiao Y.Z., Liang X.F., Jin N., Bao J., Jiang N., Zhang X.S. (2024). Real-world data of China: Analysis of HPV vaccine coverage and post-vaccination adverse reaction monitoring in Western Chinese provinces from 2018 to 2021. Hum. Vaccin. Immunother..

[37] Bruni L., Diaz M., Barrionuevo-Rosas L., Herrero R., Bray F., Bosch F.X., de Sanjose S., Castellsague X. (2016). Global estimates of human papillomavirus vaccination coverage by region and income level: A pooled analysis. Lancet Glob. Health.

[38] FENG X.-j., HOU H.-l., YU Q., WANG J.-s. (2020). Market analysis and countermeasures of cervical cancer vaccine in China. China Biotechnol..

[39] You T., Zhao X., Pan C., Gao M., Hu S., Liu Y., Zhang Y., Qiao Y., Zhao F., Jit M. (2024). Informing HPV vaccine pricing for government-funded vaccination in mainland China: A modelling study. Lancet Reg. Health West. Pac..

[40] Zou Z., Fairley C.K., Ong J.J., Shen M., Chow E.P.F., Liu H., Xia R., Li R., Hocking J., Zhuang G. (2024). Impact of achieving WHO’s 90-70-90 targets on cervical cancer elimination and potential benefits in preventing other HPV-related cancers in China: A modelling study. eClinicalMedicine.

[41] Peerenboom R., Ackroyd S., Lee N. (2024). The burden of cervical cancer survivorship: Understanding morbidity and survivorship needs through hospital admissions. Gynecol. Oncol. Rep..

[42] Tao S., Peng J., Wang Y., Zhang G., Chen Z., Zhao F., Ma J., Yang X., Qiao Y., Zhao F. (2018). Study on direct economic burden and influencing factors in patients with cervical cancer and precancerous lesions. Chin. J. Prev. Med..

[43] van Ooijen P.M.A., Cai H., Wang H., Guo T., Bao G. (2016). Application of Telemedicine in Gansu Province of China. PLoS ONE.

[44] Tudor Hart J. (1971). The inverse care law. Lancet.

[45] Aimin W., Chaojin C., Mujun W., Ruhao W., Jingjing L. (2024). Medical Expenses for Hospitalized Patients with Cervical Cancer Before and After the Implementation of the DRG Payment Policy. Med. J. Peking Union Med. Coll. Hosp..

