# Supplementary material for: Study on the Trend of Cervical Cancer Inpatient Costs and Its Influencing Factors in Economically Underdeveloped Areas of China, 2019–2023: An Analysis in Gansu Province
Source: Healthcare (Basel). 2025 Oct 22;13(21):2663. doi: 10.3390/healthcare13212663 (PMC12607449; doi:10.3390/healthcare13212663)
Supplement: Supplementary file 1 [file healthcare-13-02663-s001.zip › healthcare-3726850-supplementary.pdf]

Supplementary Material

1 Supplementary Tables

Supplemental Table S1: ICD-10 codes for Cervical cancer comorbidities.

Comorbidities	ICD-10
Cervical cancer	C53
Neurological disease	G00-G99
Endocrine disease	E00-E90
Pulmonary disease	J00-J99 (except J00-06 & J30-39)
Psychiatric disease	F00-F99
Other neoplasms	C00-D48(except C53)

Supplemental Table S2: Variable assignment of quantile regression and random forest model.

Variables	Variable assignment
Age(years)	Actual value
Payment method	1 = Medical insurance reimbursement, 2 = Out-of-pocket
Hospital nature	1 = Public, 2 = Private
Hospital level	1 = Provincial<br>2 = Municipal level<br>3 = District level<br>4 = County level
Hospital type	1 = General hospital<br>2 = TCM hospital<br>3 =Maternal and Child Healthcare Hospital<br>4 =Specialized hospital
Length of stay (days)	Actual value
Proportion of medications (%)	Actual value
Number of comorbidities	0 = 0, 1 = 1, 2 = 2, 3 = 3 or more
Cervical cancer surgery	0 = No, 1 = Yes
Year[a]	1 = 2019, 2 = 2020, 3 = 2021, 4 = 2022, 5 = 2023
Inpatient costs per time	Ln (actual value)
Average daily inpatient costs	Ln (actual value)

[a]The year is based on the patient's discharge time in the hospital information system.

Supplemental Table S3: Basic characteristics of patients in different age groups.

Category	<40		40-50		51-60		＞60	
	n(q%)	Median (lower quartile, upper quartile) (CNY)	n(q%)	Median (lower quartile, upper quartile) (CNY)	n(q%)	Median (lower quartile, upper quartile) (CNY)	n(q%)	Median (lower quartile, upper quartile) (CNY)
All cases	1125 (11.17)	18827.59(8109.92,34423.58)	2756 (27.37)	19258.37(8037.38,34868.09)	3826 (37.99)	17607.94(7322.41,36812.16)	2363 (23.47)	13777.08(6780.49,37826.75)
Payment method								
Medical insurance reimbursement	976(86.76)	20962.04(8409.02,35028.38)	2474 (89.73)	19593.81(8113.64,35109.13)	3447 (90.10)	19172.37(7457.86,38327.19)	2154 (91.15)	14401.00(6899.81,39244.97)
Out-of-pocket	149(13.24)	11886.68(6046.60,29465.34)	283(10.27)	16927.83(7817.91,33301.38)	379(9.90)	11623.68(6474.55,27555.45)	209(41.93)	10332.24(5470.41,28792.70)
Hospital nature								
Public	1104 (98.13)	19554.71(8227.59,34762.77)	2700 (97.97)	19675.75(8156.46,34988.95)	3769 (98.51)	17694.53(7327.64,36954.39)	2281 (96.53)	13777.08(6838.01,38209.58)
Private	21(1.87)	3769.78(2784.66,10601.92)	56(2.03)	10706.66(4906.98,17275.23)	57(1.49)	15532.77(6723.82,33853.98)	82(3.47)	14152.52(5261.78,34283.97)
Hospital level								
Provincial level	905(80.44)	25335.59(11128.69,37403.74)	2059 (74.71)	25897.44(11359.29,38222.75)	2796 (73.13)	26458.20(10559.51,42084.59)	1545 (65.38)	21489.80(9460.03,44550.66)
municipal level	165(14.67)	5297.71(3278.99,11922)	579(21.01)	7656.66(4256.30,18800.89)	824(21.54)	6915.26(3900.91,19897.36)	604(25.56)	8328.34(4597.36,23475.53)
District level	33(2.93)	5986.22(3982.08,6749.81)	78(2.83)	4856.75(3928.20,10724.93)	139(3.63)	6373.95(4035.68,9897.72)	132(5.59)	6298.68(4100.65,10799.99)
county level	22(1.96)	3846.93(2643.76,5425.42)	40(1.45)	13810.21(2812.20,36681.87)	67(1.75)	2870.96(2273.09,4194.36)	82(3.47)	3286.69(2359.66,4915.63)
Hospital type								
General hospital	275(24.44)	9516.70(4861.62,34327.66)	771(28.00)	13401.30(5632.64,35748.92)	1249 (32.64)	11341.89(5325.92,34491.87)	930(39.36)	11281.71(5706.09,33885.44)

Traditional Chinese Medicine (TCM) hospital	27(2.4)	7789.33(4583.15,19579.38)	70(2.54)	6499.89(3574.79,10730.68)	108(2.82)	6179.63(3319.98,16757.00)	121(5.12)	6624.69(3002.78,12344.42)
Maternal and Child Healthcare Hospital	460(40.89)	26162.28(12722.19,34895.38)	780(28.30)	27203.10(15790.26,35106.01)	1006(26.30)	29108.13(14301.88,38704.47)	482(20.40)	25640.58(9907.34,40457.69)
Specialized Hospital	363(32.27)	15530.36(7902.56,35363.09)	1135(41.18)	16121.11(8018.61,35198.18)	1463(38.21)	16520.73(7916.07,38491.95)	830(35.12)	15650.18(7949.92,44935.9)
Length of stay (days) [mean(SD)]	14.76(12.19)		15.70(13.39)		16.33(14.69)		16.93(16.21)	
Proportion of medications (%) [mean(SD)]	20.23(15.78)		22.59(17.20)		23.08(17.61)		21.49(17.67)	
Number of comorbidities								
0	328(29.16)	8911.27(5265.50,15820.26)	978(35.49)	9658.67(5424.54,17334.73)	1004(26.24)	8248.09(4460.18,20096.29)	497(20.95)	8566.73(4528.49,23371.79)
1	196(17.42)	25646.37(9508.63,35144.14)	457(16.58)	25497.48(7908.17,37303.19)	733(19.16)	11129.18(6688.35,24456.48)	478(20.23)	9683.54(5762.39,14774)
2	169(15.02)	17345.50(8637.41,32476.25)	337(12.23)	24478.70(11264.18,38157.38)	557(14.56)	19615.25(7648.78,43786.75)	394(16.67)	17564.19(6633.22,58760.86)
≥3	432(38.40)	29446.19(15634.46,41250.69)	984(35.70)	30615.14(17747.70,42992.38)	1532(40.04)	31275.35(16474.37,45187.33)	994(42.15)	26846.84(9923.41,46114.77)
Cervical cancer surgery								
No	396(35.20)	7816.96(4620.18,14186.04)	1282(46.51)	8694.65(4946.17,16927.83)	2024(52.80)	8447.30(4822.19,15558.04)	1492(63.14)	8571.55(5228.41,18134.19)
Yes	729(64.80)	26649.86(14212.39,36496.45)	1474(53.49)	28690.22(18373.69,37822.80)	1802(47.20)	31372.07(22094.19,42097.92)	871(36.86)	31964.02(18541.81,44933.90)

Supplemental Table S4: Basic characteristics of cervical cancer surgery patients and non-surgery procedure patients.

Category	Cervical cancer surgery patients		Non-surgery patients	
	n(q%)	Median (lower quartile, upper quartile) (CNY)	n(q%)	Median (lower quartile, upper quartile) (CNY)
All cases	4876(48.42)	31071.38(16827.06,45325.44)	5194(51.58)	5885.11(2955.57,11589.50)
Age(years) [mean (SD)]	51.28(10.47)		55.18(10.83)	
<40	729(14.95)	26649.86(14212.39,36496.45)	396(7.62)	7816.96(4620.18,14186.04)
40-50	1474(30.23)	28690.22(18373.69,37822.80)	1282(24.68)	8694.65(4946.16,16927.83)
51-60	1802(36.96)	31372.07(22094.19,42097.92)	2024(38.97)	8447.30(4822.18,15558.04)
＞60	871(17.86)	31964.02(18541.81,44933.90)	1492(28.73)	8571.54(5228.41,18134.19)
Payment method				
Medical insurance reimbursement	4462(91.51)	30061.69(18940.74,40509.40)	4588(88.33)	8611.15(5015.72,17664.94)
Out-of-pocket	414(8.49)	29074.24(17485.95,36983.80)	606(11.67)	7797.98(4586.37,12390.86)
Hospital nature				
Public	4769(97.81)	30058.41(19206.78,40453.75)	5085(97.90)	8531.24(5000.02,16566.18)
Private	107(2.19)	19135.48(9247.66,34283.97)	109(2.10)	6688.81(3760.16,15580.04)
Hospital level				
Provincial level	4106(84.21)	31877.63(23474.62,42049.47)	3199(61.59)	10617.97(6894.13,22023.97)
Municipal level	659(13.52)	15895.77(7862.81,25349.00)	1513(29.13)	5684.00(3508.62,11341.3)
District level	74(1.52)	16002.96(5557.76,21818.47)	308(5.93)	5480.45(3898.98,8172.43)
County level	37(0.76)	5057.00(3267.48,7433.80)	174(3.35)	3152.30(2324.26,4197.29)
Hospital type				
General hospital	1111(22.79)	34762.33(16527.92,47352.33)	2114(40.70)	7329.47(4260.53,14456.90)
Traditional Chinese Medicine (TCM) hospital	105(2.15)	10226.30(3692.91,20497.49)	221(4.25)	5668.05(3046.48,9984.19)
Maternal and Child Healthcare Hospital	2288(46.92)	30043.00(20459.26,39120.66)	440(8.47)	7099.55(5333.50,9560.07)
Specialized Hospital	1372(28.14)	29099.44(20970.70,36359.97)	2419(46.57)	10191.90(6048.95,56563.59)
Length of stay (days) [mean (SD)]	18.28(12.54)		14.09(15.83)	
Proportion of medications (%) [mean (SD)]	18.08(10.83)		26.16(21.01)	
Number of comorbidities				
0	897(18.40)	18330.30(10558.14,27240.67)	1910(36.77)	6638.64(3928.20,11168.31)
1	677(13.88)	29079.98(13787.73,37688.28)	1187(22.85)	9003.06(5325.92,13704.47)
2	635(13.02)	28374.55(16340.68,38065.70)	822(15.83)	11097.19(5820.84,60981.23)

≥3	2667(54.70)	33378.00(25840.64,43979.89)	1275(24.55)	10922.67(6333.39,55427.52)

Supplemental Table S5: Quantile Regression Results for Inpatient costs per hospitalization (Q10)

Variables	Inpatient costs per hospitalization (Q10)				
	Coefficient	Std. err.	t	P	[95% conf. interval]	
Age (contrast = <40)						
40-50	0.101	0.036	20.80	0.005	0.030	0.171
51-60	0.061	0.034	1.75	0.008	-0.007	0.129
>60	0.081	0.037	20.17	0.030	0.008	0.155
Payment method (contrast = medical insurance reimbursement)						
Out-of-pocket	-0.162	0.034	-4.75	0.000	-0.229	-0.095
Hospital nature (contrast = public)						
Private	-0.262	0.074	-3.52	0.000	-0.407	-0.116
Hospital level (contrast = provincial level)						
Municipal level	-0.525	0.030	-17.29	0.000	-0.585	-0.465
District level	-0.578	0.056	-10.17	0.000	-0.689	-0.466
county level	-0.882	0.074	-11.84	0.000	-1.027	-0.735
Hospital type (contrast = general hospital)						
Traditional Chinese Medicine (TCM) hospital	-0.479	0.060	-7.97	0.000	-0.597	-0.361
Maternal and Child Healthcare Hospital	-0.104	0.032	-3.20	0.001	-0.167	-0.040
Specialized Hospital	-0.017	0.025	-0.68	0.047	-0.048	0.033
Number of comorbidities (contrast = 0)						
1	0.065	0.032	2.04	0.041	0.003	0.129
2	0.198	0.035	5.59	0.000	0.129	0.268
≥3	0.304	0.030	9.95	0.000	0.244	0.364
Length of stay	0.046	0.001	60.18	0.000	0.044	0.047

Proportion of medications	0.758	0.065	11.60	0.000	0.630	0.886
Cervical cancer surgery(contrast = No)						
Yes	0.726	0.024	30.93	0.000	0.682	0.775
Year (contrast = 2019)						
2020	0.082	0.030	2.71	0.007	0.023	0.142
2021	0.122	0.032	3.79	0.000	0.059	0.186
2022	-0.146	0.037	-3.96	0.000	-0.219	-0.074
2023	-0.078	0.036	-2.14	0.032	-0.148	-0.007
_cons	7.826	0.053	146.40	0.000	7.721	7.931
Pseudo R^2			0.501			

Supplemental Table S6: Quantile Regression Results for Inpatient costs per hospitalization (Q50)

Variables	Inpatient costs per hospitalization (Q50)					
	Coefficient	Std. err.	t	P	[95% conf. interval]	
Age (contrast = <40)						
40-50	0.022	0.019	1.15	0.250	-0.015	0.060
51-60	0.015	0.018	0.82	0.413	-0.021	0.051
>60	0.008	0.020	0.42	0.677	-0.031	0.048
Payment method (contrast = medical insurance reimbursement)						
Out-of-pocket	-0.044	0.018	-2.41	0.016	-0.079	-0.008
Hospital nature (contrast = public)						
Private	0.034	0.039	0.85	0.396	-0.044	0.111
Hospital level (contrast = provincial level)						
Municipal level	-0.514	0.016	-31.83	0.000	-0.545	-0.482
District level	-0.527	0.030	-17.46	0.000	-0.586	-0.468
county level	-1.067	0.040	-27.00	0.000	-1.145	-0.990
Hospital type (contrast = general hospital)						
Traditional Chinese	-0.282	0.032	-7.97	0.000	-0.597	-0.361

Medicine (TCM) hospital						
Maternal and Child Healthcare Hospital	-0.159	0.017	-9.24	0.000	-0.193	-0.126
Specialized Hospital	-0.066	0.014	-4.79	0.000	-0.093	-0.039
Number of comorbidities (contrast = 0)						
1	0.096	0.017	5.63	0.000	0.063	0.129
2	0.119	0.019	6.34	0.000	0.082	0.156
≥3	0.165	0.016	10.15	0.000	0.133	0.196
Length of stay	0.052	0.000	128.61	0.000	0.051	0.053
Proportion of medications	0.356	0.035	10.25	0.000	0.288	0.424
Cervical cancer surgery(contrast = No)						
Yes	0.698	0.013	55.84	0.000	0.674	0.723
Year (contrast = 2019)						
2020	0.055	0.016	3.40	0.001	0.023	0.086
2021	0.037	0.017	2.16	0.030	0.004	0.071
2022	-0.035	0.020	-1.78	0.075	-0.073	0.004
2023	0.011	0.019	0.59	0.558	-0.026	0.049
_cons	8.597	0.028	302.96	0.000	8.541	8.652
Pseudo R^2			0.586			

Supplemental Table S7: Quantile Regression Results for Inpatient costs per hospitalization (Q90)

Variables	Inpatient costs per hospitalization (Q90)					
	Coefficient	Std. err.	t	P	[95% conf. interval]	
Age (contrast = <40)						
40-50	0.022	0.029	0.77	0.440	-0.034	0.079
51-60	0.007	0.028	0.25	0.805	-0.048	0.062
>60	0.026	0.030	0.85	0.397	-0.034	0.085
Payment method (contrast = medical insurance reimbursement)						

Out-of-pocket	0.009	0.027	0.31	0.041	-0.045	0.062
Hospital nature (contrast = public)						
Private	0.206	0.060	3.45	0.001	0.089	0.323
Hospital level (contrast = provincial level)						
Municipal level	-0.299	0.024	-12.27	0.000	-0.347	-0.251
District level	-0.694	0.046	-15.19	0.000	-0.783	-0.604
county level	-1.185	0.060	-19.82	0.000	-1.302	-1.068
Hospital type (contrast = general hospital)						
Traditional Chinese Medicine (TCM) hospital	-0.350	0.048	-7.26	0.000	-0.445	-0.256
Maternal and Child Healthcare Hospital	-0.349	0.026	-13.37	0.000	-0.400	-0.297
Specialized Hospital	-0.235	0.021	-11.33	0.000	-0.276	-0.195
Number of comorbidities (contrast = 0)						
1	0.147	0.026	5.70	0.000	0.096	0.197
2	0.158	0.028	5.55	0.000	0.102	0.214
≥3	0.177	0.025	7.20	0.000	0.129	0.225
Length of stay	0.053	0.001	86.71	0.000	0.052	0.054
Proportion of medications	0.051	0.052	0.98	0.329	-0.052	0.154
Cervical cancer surgery(contrast = No)						
Yes	0.521	0.019	27.52	0.000	0.484	0.558
Year (contrast = 2019)						
2020	0.028	0.024	1.16	0.244	-0.019	0.076
2021	0.045	0.026	1.75	0.081	-0.006	0.096
2022	-0.006	0.030	-0.20	0.845	-0.064	0.052
2023	0.009	0.029	0.30	0.764	-0.048	0.066
_cons	9.306	0.043	216.79	0.000	9.222	9.390
Pseudo R^2		0.537				

Supplemental Table S8: Quantile Regression Results for Average Daily Inpatient Cost (Q10)

Variables	Average daily hospitalization costs (Q10)					
	Coefficient	Std. err.	t	P	[95% conf. interval]	
Age (contrast = <40)						
40-50	0.059	0.030	1.95	0.052	0.000	0.118
51-60	0.035	0.029	1.19	0.233	-0.022	0.092
>60	0.048	0.032	1.52	0.129	-0.014	0.110
Payment method (contrast = medical insurance reimbursement)						
Out-of-pocket	-0.090	0.029	-3.14	0.002	-0.146	-0.034
Hospital nature (contrast = public)						
Private	-0.072	0.062	-1.16	0.244	-0.194	0.049
Hospital level (contrast = provincial level)						
Municipal level	-0.567	0.025	-22.32	0.000	-0.617	-0.517
District level	-0.631	0.048	-13.26	0.000	-0.724	-0.537
county level	-0.929	0.062	-14.92	0.000	-1.051	-0.807
Hospital type (contrast = general hospital)						
Traditional Chinese Medicine (TCM) hospital	-0.227	0.050	-4.51	0.000	-0.325	-0.128
Maternal and Child Healthcare Hospital	-0.015	0.027	-0.53	0.593	-0.068	0.039
Specialized Hospital	-0.026	0.022	-1.20	0.230	-0.068	0.016
Number of comorbidities (contrast = 0)						
1	0.031	0.027	1.15	0.025	-0.022	0.083
2	0.093	0.030	3.14	0.002	0.035	0.151
≥3	0.129	0.026	5.05	0.000	0.079	0.179
Length of stay	0.004	0.001	5.96	0.000	0.003	0.005
Proportion of medications	0.275	0.055	5.04	0.000	0.168	0.383

Cervical cancer surgery(contrast = No)						
Yes	0.395	0.020	20.06	0.000	0.357	0.434
Year (contrast = 2019)						
2020	0.020	0.025	0.77	0.440	-0.030	0.069
2021	0.016	0.027	0.61	0.543	-0.037	0.069
2022	-0.068	0.031	-2.21	0.027	-0.129	-0.008
2023	-0.010	0.030	-0.35	0.730	-0.070	0.049
_cons	6.532	0.045	146.08	0.000	6.444	6.620
Pseudo R^2		0.298				

Supplemental Table S9: Quantile Regression Results for Average Daily Inpatient Cost (Q50)

Variables	Average daily hospitalization costs (Q50)					
	Coefficient	Std. err.	t	P	[95% conf. interval]	
Age (contrast = <40)						
40-50	0.008	0.018	0.46	0.646	-0.027	0.043
51-60	0.008	0.017	0.47	0.640	-0.026	0.042
>60	0.011	0.019	0.57	0.569	-0.026	0.047
Payment method (contrast = medical insurance reimbursement)						
Out-of-pocket	-0.053	0.017	-3.15	0.002	-0.087	-0.020
Hospital nature (contrast = public)						
Private	0.049	0.037	1.33	0.184	-0.023	0.122
Hospital level (contrast = provincial level)						
Municipal level	-0.443	0.015	-29.31	0.000	-0.472	-0.413
District level	-0.567	0.028	-20.05	0.000	-0.622	-0.511
county level	-0.941	0.037	-25.43	0.000	-1.013	-0.868
Hospital type (contrast = general hospital)						
Traditional Chinese Medicine (TCM) hospital	-0.327	0.030	-10.96	0.000	-0.386	-0.269

Maternal and Child Healthcare Hospital	-0.159	0.016	-9.83	0.000	-0.190	-0.127
Specialized Hospital	-0.106	0.013	-8.28	0.000	-0.132	-0.081
Number of comorbidities (contrast = 0)						
1	0.067	0.016	4.21	0.000	0.036	0.098
2	0.102	0.018	5.77	0.000	0.067	0.136
≥3	0.106	0.015	6.99	0.000	0.076	0.136
Length of stay	0.001	0.000	3.47	0.001	0.001	0.002
Proportion of medications	-0.036	0.032	-1.12	0.263	-0.100	0.027
Cervical cancer surgery(contrast = No)						
Yes	0.292	0.012	24.98	0.000	0.269	0.315
Year (contrast = 2019)						
2020	0.075	0.015	4.95	0.000	0.045	0.104
2021	0.037	0.016	2.30	0.021	0.006	0.068
2022	-0.030	0.018	-1.61	0.107	-0.066	0.006
2023	0.019	0.018	1.05	0.293	-0.016	0.054
_cons	7.234	0.027	272.35	0.000	7.182	7.286
Pseudo R^2			0.208			

Supplemental Table S10: Quantile Regression Results for Average Daily Inpatient Cost (Q90)

Variables	Average daily hospitalization costs (Q90)					
	Coefficient	Std. err.	t	P	[95% conf. interval]	
Age (contrast = <40)						
40-50	0.008	0.027	0.31	0.760	-0.045	0.062
51-60	-0.001	0.026	-0.02	0.983	-0.052	0.051
>60	0.025	0.028	0.88	0.376	-0.031	0.081
Payment method (contrast = medical insurance reimbursement)						

Out-of-pocket	-0.007	0.026	-0.27	0.784	-0.058	0.043
Hospital nature (contrast = public)						
Private	0.243	0.056	4.33	0.000	0.133	0.353
Hospital level (contrast = provincial level)						
Municipal level	-0.328	0.023	-14.31	0.000	-0.373	-0.283
District level	-0.714	0.043	-16.63	0.000	-0.799	-0.630
county level	-0.856	0.056	-15.22	0.000	-0.966	-0.746
Hospital type (contrast = general hospital)						
Traditional Chinese Medicine (TCM) hospital	-0.459	0.045	-10.10	0.000	-0.548	-0.370
Maternal and Child Healthcare Hospital	-0.324	0.025	-13.20	0.000	-0.372	-0.276
Specialized Hospital	-0.290	0.020	-14.82	0.000	-0.328	-0.252
Number of comorbidities (contrast = 0)						
1	0.115	0.024	4.74	0.000	0.067	0.162
2	0.075	0.027	2.81	0.005	0.023	0.128
≥3	0.090	0.023	3.89	0.000	0.044	0.135
Length of stay	-0.005	0.001	-9.17	0.000	-0.006	-0.004
Proportion of medications	-0.145	0.049	-2.93	0.003	-0.241	-0.048
Cervical cancer surgery(contrast = No)						
Yes	0.092	0.018	5.17	0.000	0.057	0.127
Year (contrast = 2019)						
2020	0.039	0.023	1.69	0.091	-0.006	0.084
2021	0.042	0.024	1.72	0.086	-0.006	0.090
2022	-0.014	0.028	-0.52	0.605	-0.069	0.040
2023	-0.006	0.027	-0.21	0.833	-0.059	0.048
_cons	8.089	0.040	200.36	0.000	8.010	8.168
Pseudo R^2		0.122				

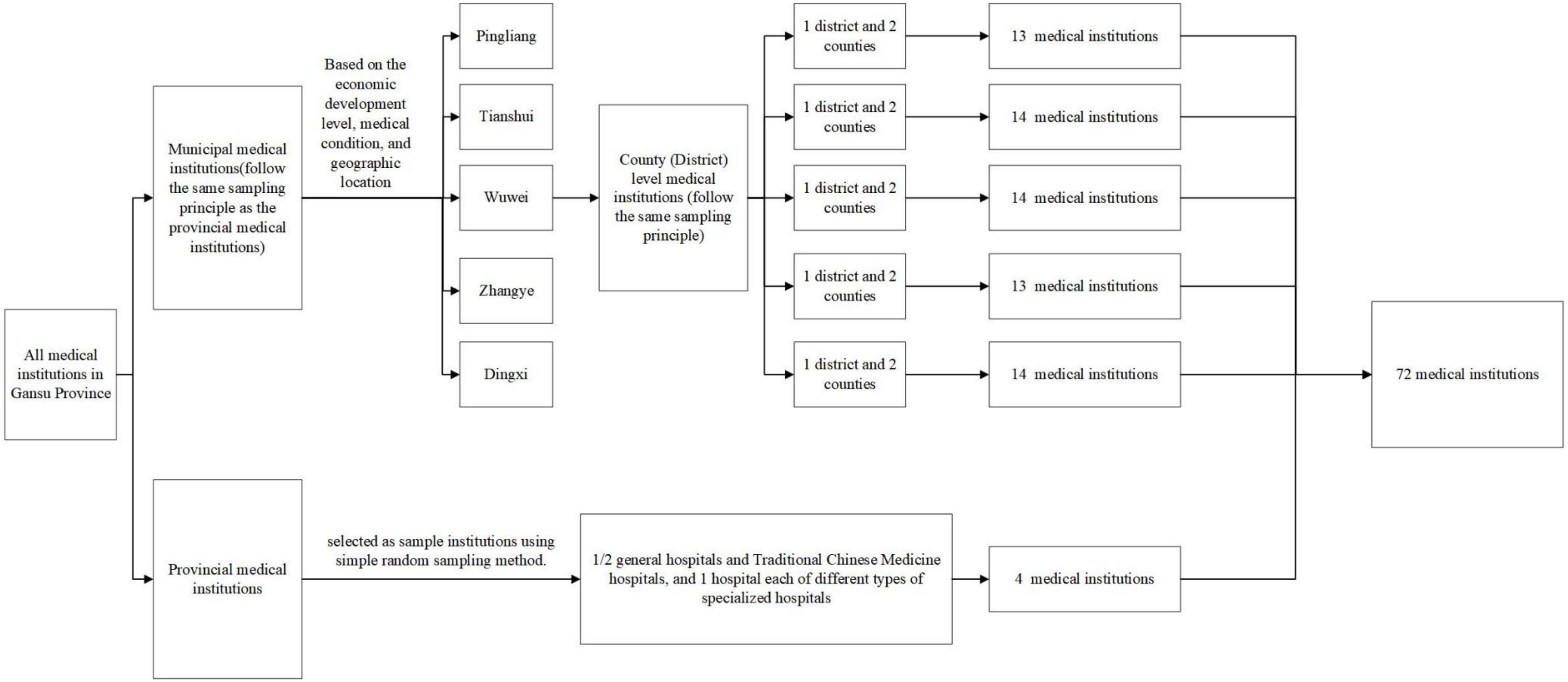

Supplementary Figure S1 Institutional selection process flowchart. Medical institutions were sampled stratified by administrative level. Provincial medical institutions pertain to the administrative level of provinces, municipal medical institutions align with municipal administrative levels, and district (county) medical institutions correspond to the district (county) administrative levels.

Note: The reason why Pingliang and Zhangye only have 13 medical institutions is that their specialized hospitals are rheumatology specialized hospitals and orthopedics specialized hospitals respectively. No inpatient information with the primary diagnosis of cervical cancer was found in these specialized hospitals, so the number of medical institutions included in the final statistics is 13 for these two regions.

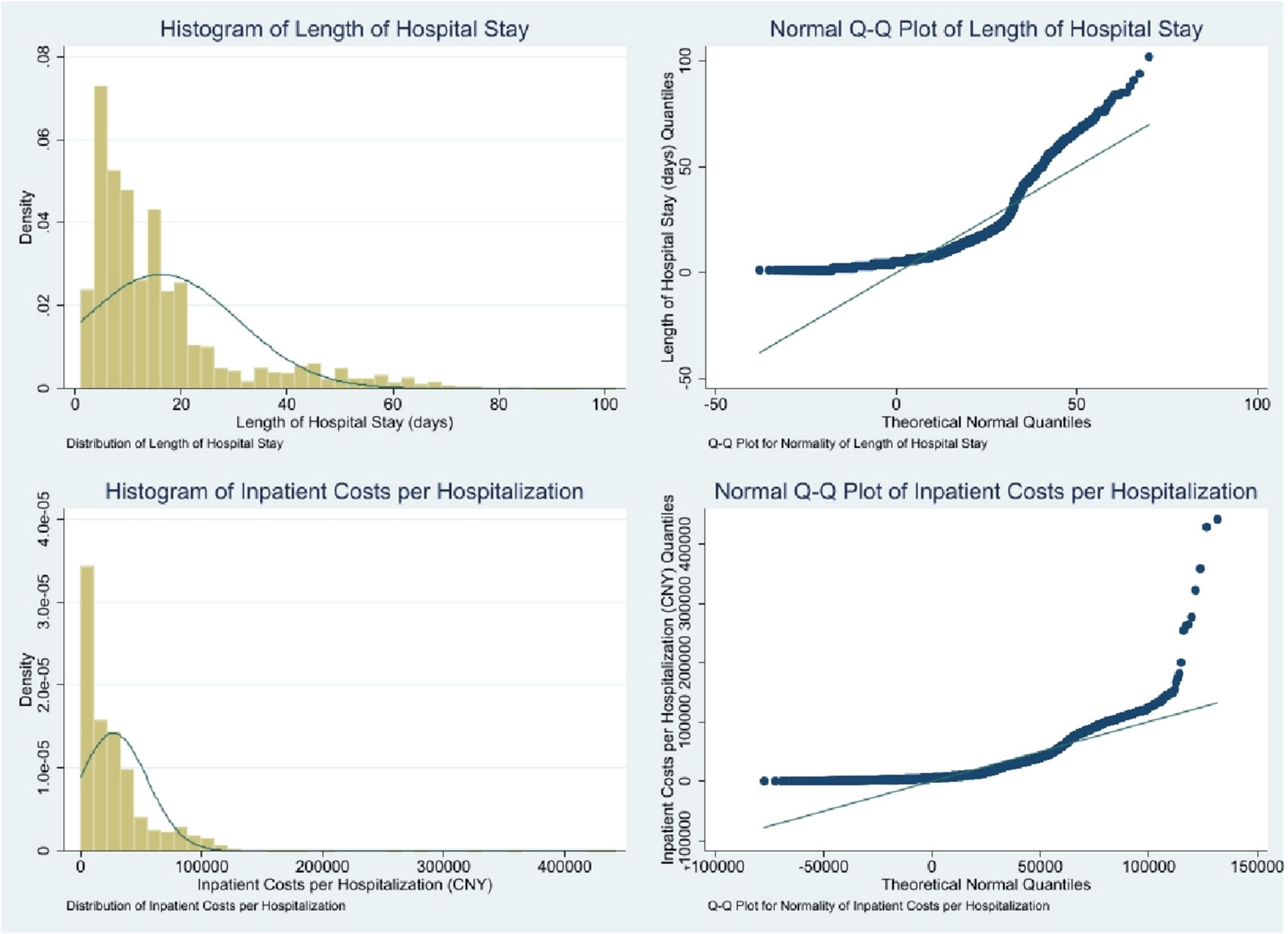

Supplementary Figure S2 Distribution of inpatient costs and length of hospital stay. Histograms and Q – Q plots indicated right-skewed distributions for both variables, supporting the use of non-parametric tests in univariable analyses.
